# A Negative Index Metamaterial-Inspired UWB Antenna with an Integration of Complementary SRR and CLS Unit Cells for Microwave Imaging Sensor Applications

**DOI:** 10.3390/s150511601

**Published:** 2015-05-20

**Authors:** Mohammad Tariqul Islam, Md. Moinul Islam, Md. Samsuzzaman, Mohammad Rashed Iqbal Faruque, Norbahiah Misran

**Affiliations:** 1 Department of Electrical, Electronic and Systems Engineering, Faculty of Engineering and Built Environment, Universiti Kebangsaan Malaysia, Bangi, 43600 Selangor, Malaysia; E-Mails: sobuzcse@eng.ukm.my (M.S.); bahiah@eng.ukm.my (N.M.); 2 Space Science Centre (ANGKASA), Research Centre Building, Universiti Kebangsaan Malaysia, Bangi, 43600 Selangor, Malaysia; E-Mails: mmoiislam@siswa.ukm.edu.my (M.M.I.); rashed@ukm.edu.my (M.R.I.F.)

**Keywords:** microwave imaging, metamaterial, sensor, ultra-wideband

## Abstract

This paper presents a negative index metamaterial incorporated UWB antenna with an integration of complementary SRR (split-ring resonator) and CLS (capacitive loaded strip) unit cells for microwave imaging sensor applications. This metamaterial UWB antenna sensor consists of four unit cells along one axis, where each unit cell incorporates a complementary SRR and CLS pair. This integration enables a design layout that allows both a negative value of permittivity and a negative value of permeability simultaneous, resulting in a durable negative index to enhance the antenna sensor performance for microwave imaging sensor applications. The proposed MTM antenna sensor was designed and fabricated on an FR4 substrate having a thickness of 1.6 mm and a dielectric constant of 4.6. The electrical dimensions of this antenna sensor are 0.20 λ × 0.29 λ at a lower frequency of 3.1 GHz. This antenna sensor achieves a 131.5% bandwidth (VSWR < 2) covering the frequency bands from 3.1 GHz to more than 15 GHz with a maximum gain of 6.57 dBi. High fidelity factor and gain, smooth surface-current distribution and nearly omni-directional radiation patterns with low cross-polarization confirm that the proposed negative index UWB antenna is a promising entrant in the field of microwave imaging sensors.

## 1. Introduction

At present, microwave imaging sensors are widely used in medical imaging applications such as breast cancer, heart failure and brain stroke detection. Aiming to detect unwanted cells, an imaging system often consists of a circular cylindrical array [1] of antenna sensors. There has been huge interest in using microwave imaging antenna sensors for medical imaging owing to the fact that UWB signals offer good penetration and resolution properties. A tapered slot UWB antenna was proposed as a sensor for microwave breast imaging [2]. A negative-index metamaterial can be defined as a material with an engineered structure exhibiting electromagnetic properties not usually found in Nature. These metamaterials can exhibit some extraordinary characteristics such as simultaneous negative permittivity and permeability over a specific frequency range and negative refractive index. Metamaterials with simultaneous negative permittivity (ɛ) and permeability (µ) are also called double negative (DNG) metamaterials, left-handed metamaterials (LHM), or negative index metamaterials. Metamaterials have generated great expectations for developing efficient microwave devices, such as antenna sensors, and are leading a renaissance in microwave imaging. In 1968, Russian scientist Veselago theoretically predicted the feasibility of engineering a material that could simultaneously possess both permeability and negative permittivity [3]. Pendry made metamaterials in 1999 with SRR where the EM (electromagnetic) wave was guided along a path that was contrary to the conventional route [4]. Finally in 2000, Smith *et al.* [5] successfully constructed an artificial material of having both negative ɛ and negative µ realizing and validating the concept. Due to miniaturization, cost effectiveness and the capabilities of label-free detection, recent research has been focused on designing microwave antenna sensors based on metamaterials. Rusni *et al.* [6] reviewed multi-ring with multiple gap rectangular SRRs onto a microstrip transmission line. Yang *et al.* [7] reviewed the performance of microwave sensors using metamaterials. Chen *et al.* [8] proposed metamaterial applications in sensing with emphasis on SRR-based sensors. Sensors have been effectively introduced to displacement and velocity detection based on modified SRRs [9]. Using various types of structures, different types of artificial negative index metamaterials have been proposed, such as SRRs [10], complementary electric field-coupled resonators (CLECs) [11], spiral resonators [12], broad-side-coupled SRRs [13], fishnet structures [14], cut wire pairs [15], double-sided SRRs [16], SRR pairs [17], double-bowknot shaped resonators [18], H-shaped pairs of periodic arrays [19] and transmission-line based structures [20]. The study of negative index metamaterials has been enhanced through various strategies and processes. However, some difficulties have been encountered. These materials are difficult to fabricate and use, especially for antenna sensor design and manufacture. Their narrow bandwidths also limit the spectrum and range of their applications. Therefore, there is a need for research into addressing these drawbacks and widening the application of antenna sensing using metamaterials.

Microstrip patch antennas have been proposed for sensing applications [21,22]. Abbosh reviewed the performance of an elliptical tapered slot antenna for UWB medical imaging [23] whose electrical dimensions were 0.52 λ × 0.52 λ at a lower frequency of 3.10 GHz. This antenna covered the frequency bands from 3.1 to 11 GHz with a fractional bandwidth of 112.01%, but the antenna dimensions (50 mm × 50 mm) were too large. Majid *et al.* [24] studied an LHM structure that was designed to increase the gain of a microstrip antenna. The performance of the microstrip antenna has been investigated where the LHM structures were located in front of the patch antenna. Directional and higher gain properties were observed because of the placement of the LHM. But, the dimensions of this design architecture were too large. Alhawari *et al.* [25] reviewed an UWB antenna with a negative index metamaterial. This antenna covered the frequency range from 5.2 to 13.9 GHz with a directivity of 1.95–5.45 dB and an optimum gain of 1.2–3.85 dBi. The metamaterial used was effectively compatible with the production of negative index materials at low cost. However, the electrical dimensions of this antenna were too large (0.43 λ × 0.43 λ) and resulted in small gain and directivity, and the incomplete UWB band. Kanj *et al.* [26] observes a microstrip-fed “Dark Eyes” antenna for near-field microwave sensing having electrical dimensions 0.20 λ × 0.18 λ at a lower frequency of 2.70 GHz. The impedance bandwidth covered 2.70–9.70 GHz with a fractional bandwidth of 112.90%. The overall dimensions were 22.25 mm × 20 mm. The gain was not reported. Nordin *et al.* [27] investigated an UWB metamaterial antenna with a modified SRR and CLS where the electrical dimensions were 0.21 λ × 0.20 λ at a lower frequency of 2.90 GHz. Three unit cells were located on the patch along one axis. This antenna covered the frequency range from 2.9 to 9.9 GHz. However, the UWB band (3.1–10.6 GHz) was not completely covered. Several ultra-wideband antennas were presented with low distortion, compact size, and different shapes for microwave imaging [28,29,30]. Each antenna had its own advantages and disadvantages. Some of them lacked a planar structure, whereas others had low-gain and/or low radiation efficiency. Islam *et al.* [31] reviewed an UWB metamaterial antenna using modified SRR and CLS having electrical dimensions of 0.18 λ × 0.24 λ where 3.40 GHz was the lower frequency. The antenna’s overall size was 16 mm × 21 mm, and its gain was 1.0~5.16 dBi. It covered the frequency band from 3.40 to 12.5 GHz with a fractional bandwidth 114.50%; the UWB band (3.1–10.6 GHz) was not covered completely.

In this article, a negative index metamaterial-inspired antenna with complementary SRR and CLS for microwave imaging sensor applications has been proposed. The antenna has a compact UWB profile with high gain and fidelity factor, an omni-directional radiation pattern with low cross-polarization, and smooth surface current distribution. It consists of four MTM unit cells along one axis, including a tapered microstrip feed and a partial ground plane belonging to a rectangular slot. The proposed antenna provides an impedance bandwidth spanning from 3.1 to more than 15 GHz over the operating frequency. The electrical dimensions of this antenna sensor are 0.20 λ × 0.29 λ where the fractional bandwidth, and maximum gain are 131.5% and 6.57 dBi, respectively. Metamaterial unit cells with an integration of CSRR and CLS exhibit simultaneously negative permittivity and negative permeability, as well as negative refractive index to enhance the antenna performance for MIS applications. 

## 2. The Metamaterial Unit Cell Configuration 

### 2.1. Construction of the Structure 

**Figure 1:**
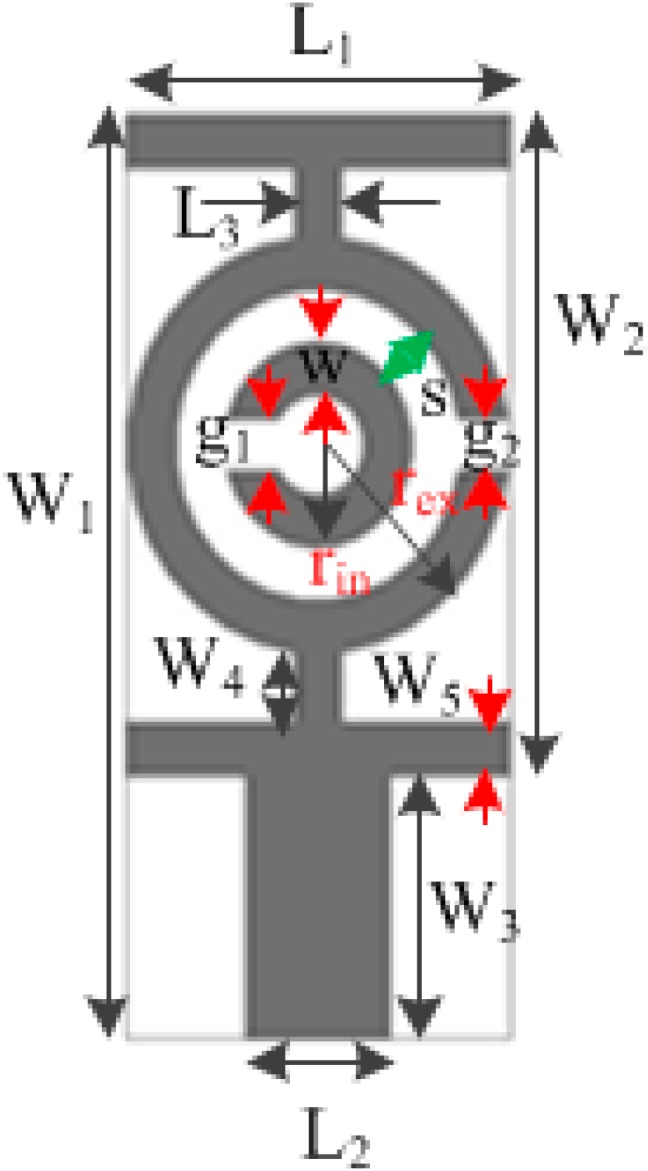
Front view of the complementary SRR unit cell with CLS.

The design of the proposed antenna begins with a study of a metamaterial unit cell. Our goal is to achieve a unit cell layout that has a resonance property that covers the operating frequency range spanning from 3.1 to 10.6 GHz. For obtaining negative values of permittivity and permeability, the well-known methods of metamaterial design are using SRRs [4,5]. The SRR was constructed using two opposing concentric split rings that are structured as two loops [4]. Due to having a magnetically resonant structure, the SRR induces a perpendicular magnetic field that is responsible for the negative value of permeability. A split gap in the inner ring introduces a capacitance that can be used to control the resonant characteristics of the structure. A complementary SRR unit cell with CLS is illustrated in Figure 1. 

**Table 1:** Design parameters of the metamaterial unit cell.

Design Parameter	Dimension (mm)	Design Parameter	Dimension (mm)
W_1_	9	L_3_	0.484
W_2_	6	g_1_	0.5
W_3_	2.64	g_2_	0.5
W_4_	0.703	s	0.5
W_5_	0.528	w	0.5
L_1_	3.872	r_in_	1
L_2_	1.452	r_ex_	2

This unit cell is printed on a 1.6 mm-thick substrate composed of FR4, a low dielectric material having a dielectric constant of 4.6. Two CLSs have been added to the upper and lower sections of the complementary SRR metamaterial unit cell, so that the characteristic resonance is obtained in the frequency range 3.1–10.6 GHz. A CLS of I-shaped strip line functions as an electric dipole and simulates a long metallic wire [25]. Because the CLS resonates through a parallel electric field and the SRR resonates through a perpendicular magnetic field, the combined layout of the complementary SRR and CLS allows for simultaneous magnetic and electric resonance [32]. The two resonance mechanism enables a lower resonance for the entire structure through the combined induced current [33]. Table 1 lists the design parameters of the proposed unit cell.

### 2.2. Simulation Setup 

**Figure 2:**
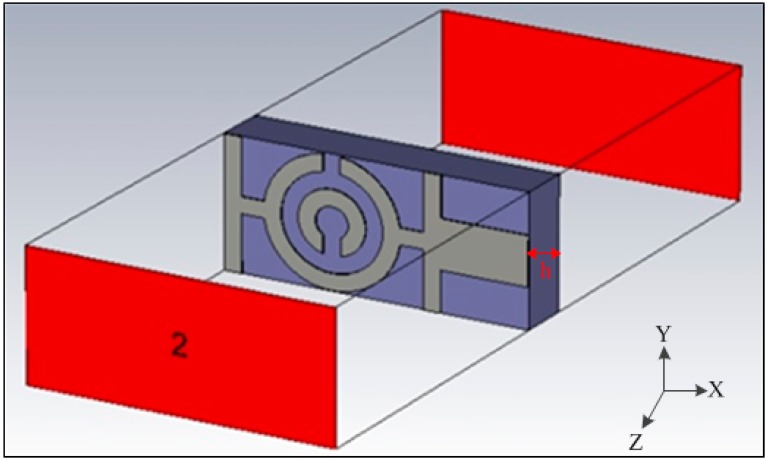
Simulation geometry of the proposed unit cell.

Using finite-difference time domain (FTTD), the MTM unit cell has been simulated based on Computer Simulation Technology (CST) software to obtain the S-parameters. The simulation geometry of the proposed unit cell is shown in Figure 2. The structure used for testing is located between two waveguide ports situated on each side of the x-axis. An electromagnetic wave was excited along the x-axis. A perfectly electrically conducting boundary condition was applied along the walls perpendicular to the y axis, and a perfectly magnetically conducting boundary was applied at the walls perpendicular to z-axis. A frequency domain solver was used to simulate the metamaterial structure. The normalized impedance was set to 50 Ω. The simulation was carried out over the frequency range spanning from 3 GHz–15 GHz.

### 2.3. Equivalent Circuit Model 

**Figure 3:**
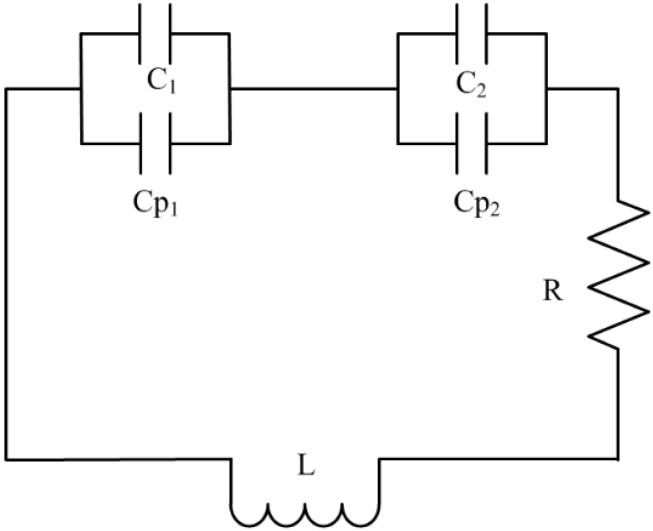
Equivalent circuit of the proposed unit cell.

The equivalent circuit model of the proposed unit cell is shown in Figure 3, where L indicates the total inductance and *C_1_* and *C_2_* are distributed capacitances which form at the two halves of the SRR structure above and below the split gaps. This circuit model also includes gap capacitances *Cp_1_* and *Cp_2_*. An electromotive force is induced around the SRR due to the application of an external magnetic field along the z-axis of the SRR. This induced electromotive force creates currents that pass from one ring to the other ring through the inter ring spacing, s and the structure acts as a LC circuit. The resonance frequency of the proposed unit cell resonator is:

(1) $$f=\frac{1}{2\pi \sqrt{LC_{P}}}$$

The total inductance, *L* of the proposed structure can be computed according to [34]:

(2) $$L=0.0002l(\log_{10}\frac{4l}{d}-\theta )$$

where *d* and *l* are the wire width and length, respectively and the constant *θ* is set equal to 2.451 [34].

The total equivalent capacitance, *C_P_* of the structure can be calculated from the equivalent circuit shown in Figure 3:

(3) $$C_{P}=\frac{(\pi r_{in}-g)C_{pul}}{2}+\frac{\varepsilon_{0}wt}{2g}$$

where, *t* and *w* are the thickness and width of the rings, respectively and $\varepsilon_{0}$ is the permittivity of free space. The split gaps dimensions are *g_1_* = *g_2_* = *g* and *r_in_* is the radius of the inner ring. $C_{pul}$ is the capacitance per unit length between the rings and is calculated as follows:

(4) $$C_{pul}=\frac{\sqrt{\varepsilon_{e}}}{c_{0}Z_{0}}$$

where $Z_{0}$ is the impedance of the medium, $c_{0}$ = 3 × 108 m/s is the velocity of the light in free space and $\varepsilon_{e}$ is the effective permittivity of the medium.

**Figure 4:**
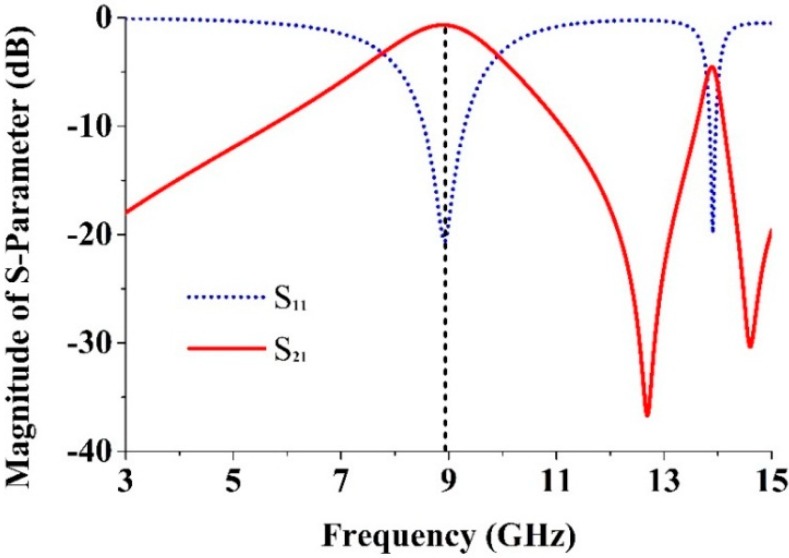
Simulated results of S-parameters for the unit cell plotted in Figure 1.

### 2.4. Retrieval of the Effective Parameters 

The S parameters such as the reflection coefficient (S_11_) and the transmission coefficient (S_21_) were obtained from the simulation and exported to the software Math CAD. A transmission peak occurs at 8.9 GHz, indicating a left-handed band (Figure 4). From the self-resonance, overlap, and the larger overall current responses with respect to existing SRRs designs, it is clear that the proposed metamaterial’s magnetic response is the main advantage. The Nicolson-Ross-Weir approach [6] was used to extract the constitutive effective parameters from S_21_ and S_11_ including the refractive index *n_r_*, the relative effective permittivity *ε_r_*, and the permeability *μ_r_*. These were obtained individually according to:

(5) $$\varepsilon_{r}=\frac{2}{jk_{0}d}*\frac{1-V_{1}}{1+V_{1}}$$

(6) $$\mu_{r}=\frac{2}{jk_{0}d}*\frac{1-V_{2}}{1+V_{2}}$$

(7) $$n_{r}=\sqrt{\varepsilon_{r}\mu_{r}}$$

(8) $$V_{1}=S_{21}+S_{11}$$

(9) $$V_{2}=S_{21}-S_{11}$$

where:

$k_{0}=\omega /c$
ω=2πf, angular frequency
d= Slab thickness
c= Speed of light

**Figure 5:**
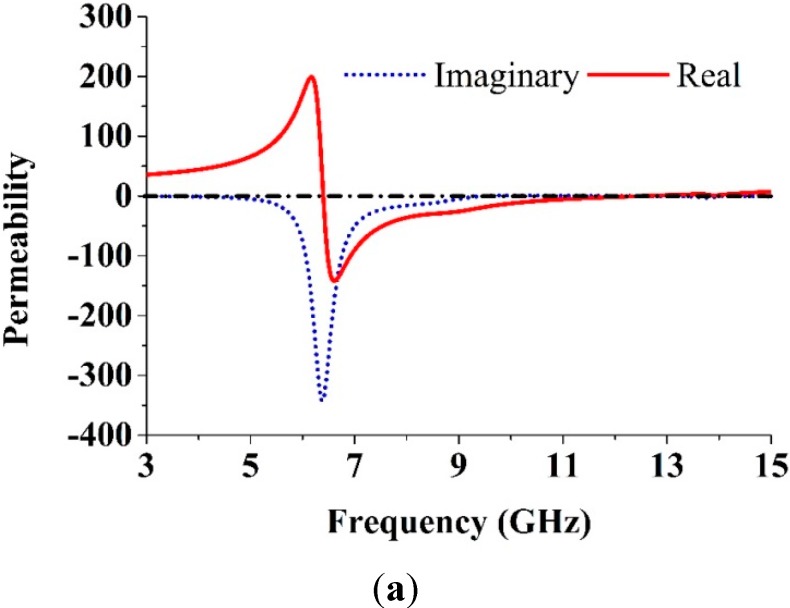

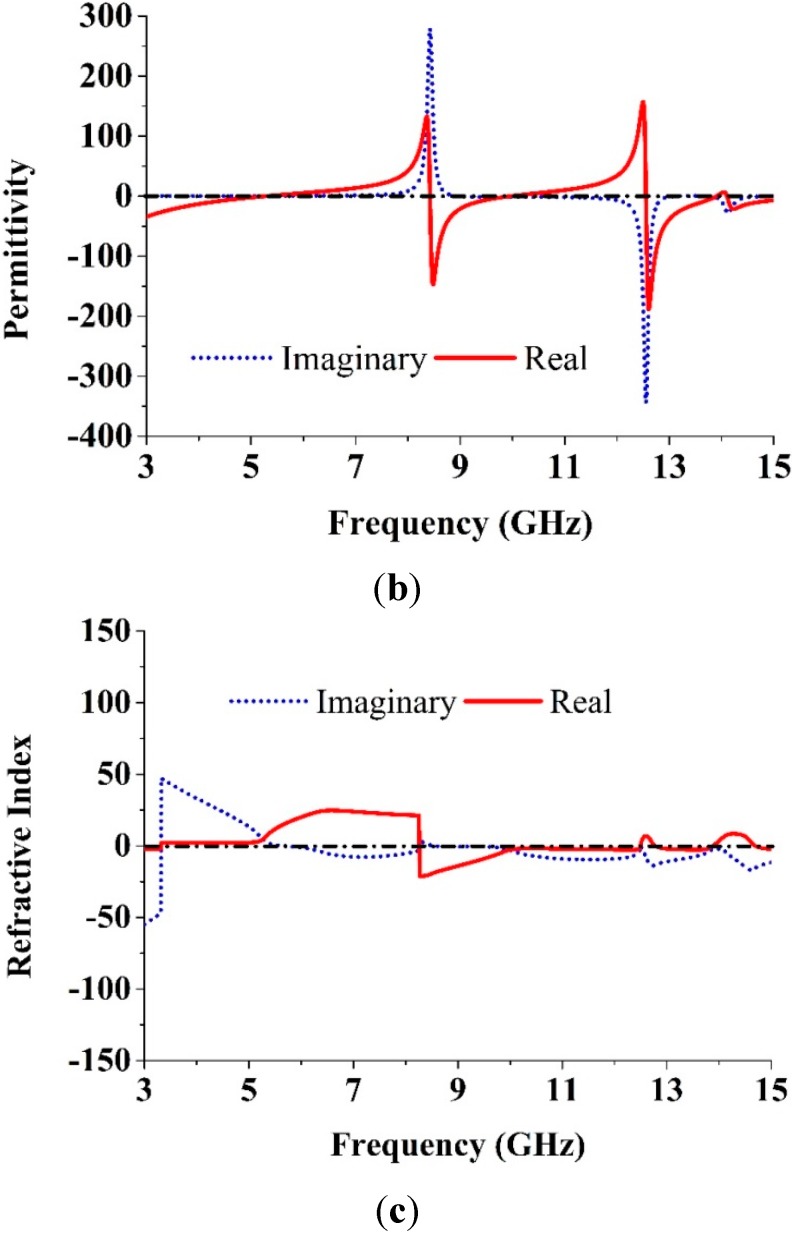
The observed effective parameters. (**a**) Permeability; (**b**) Permittivity; (**c**) Refractive index of the proposed unit cell.

Equations (5)–(9) were used for retrieving the effective parameters. Figure 5 shows the retrieved effective parameters: the refractive index, the permeability, and the permittivity of the reported MTM unit cell. The details of the negative-index frequency region are listed in Table 2. From Table 2, it can be observed that the MTM unit cell has a different resonance bandwidth in the negative-index frequency regions. This behavior indicates that the parameters of the proposed MTM structures significantly improved compared with those of previously reported MTMs that also possess negative values over a broad band [13,14,15,16,20,25,31]. 

**Table 2:** The retrieved effective parameters in the negative index frequency region.

Parameter	Negative Index Frequency Region (GHz)
Permeability, *µ_r_*	6.41–12.49
Permittivity, *ɛ_r_*	3–5.27, 8.42–9.96, 12.56–13.90, 14.11–15
Refractive index, *n_r_*	3–3.31, 8.27–12.49, 12.78–13.87,14.70–15

## 3. The MTM Antenna 

**Figure 6:**
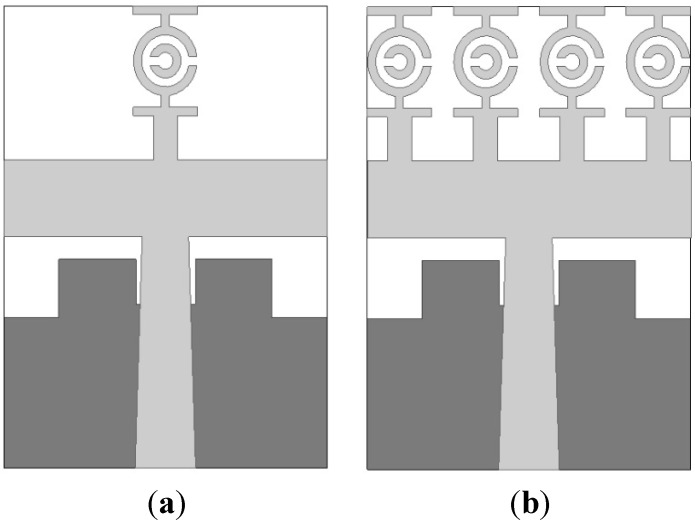
The MTM antenna (**a**) One element; (**b**) Four element.

**Figure 7:**
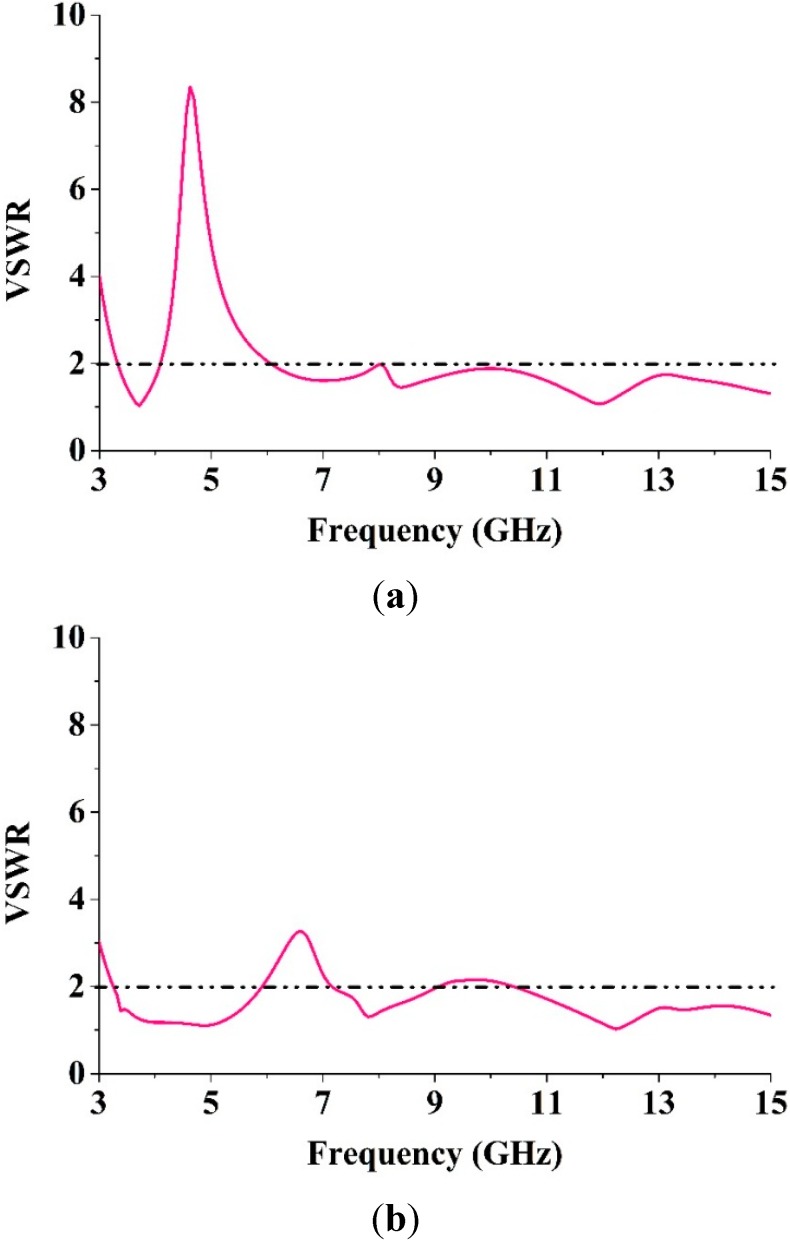
The VSWR of the MTM antenna (**a**) One element; (**b**) Four element.

The MTM antenna configurations with one and four elements are drawn in Figure 6a,b, respectively. The design layout of the proposed antenna begins with one unit cell on the rectangular resonator. The overall dimensions of this proposed antenna are 19.36 mm × 27.72 mm and a 50 Ω impedance was delivered through the port. The EM solver CST was applied for simulating the proposed antenna. The VSWR of this antenna is drawn in Figure 7 with one and four elements. It can be seen from Figure 7 that there was better matching for both one and four elements at higher frequencies. Our goal was to achieve an MTM antenna layout that has a resonance property that covers the operating frequency range spanning from 3.1 to 10.6 GHz with negative-index metamaterial properties for use in microwave imaging sensors.

## 4. UWB Metamaterial Antenna 

**Figure 8:**
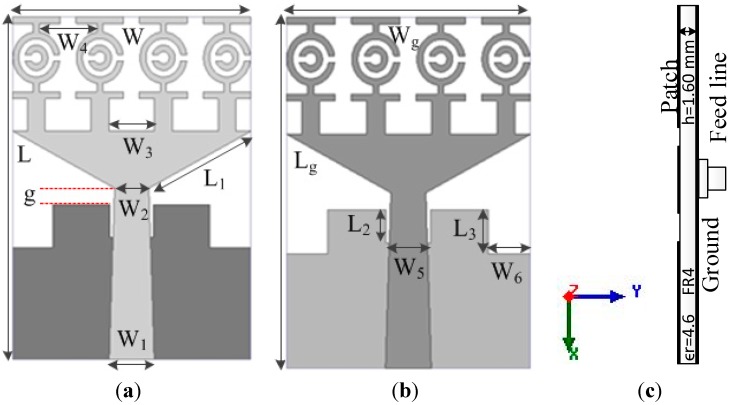
The proposed UWB antenna (**a**) Front view; (**b**) Bottom view; (**c**) Cross sectional view.

**Figure 9:**
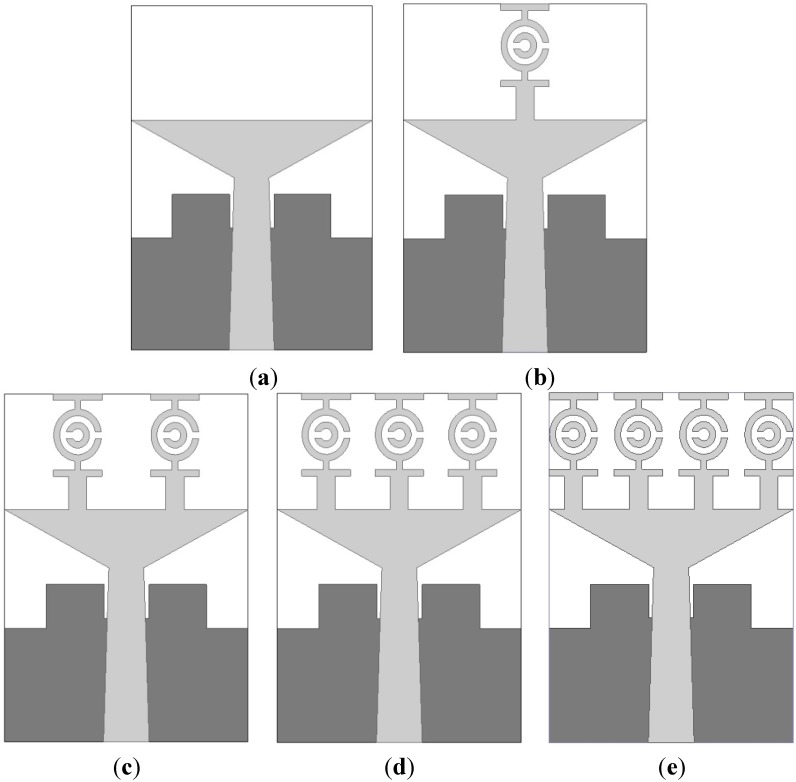
The proposed antenna. (**a**) No unit cell; (**b**) One unit cell; (**c**) Two unit cells; (**d**) Three unit cells; (**e**) Four unit cells (proposed).

Figure 8 shows the structure of the proposed UWB antenna. An FR4 substrate having a thickness of 1.6 m and a dielectric constant of 4.6 was used to print the proposed antenna. Table 3 contains the design parameters of the antenna that were found after post-optimization. This UWB antenna is made of four MTM unit cells along the y-axis including a tapered microstrip feed and a partial ground plane belonging to a rectangular slot. Each unit cell has a similar shape. Figure 9 shows the proposed UWB antenna with zero, one, two, three and four unit cells. 

**Table 3:** Antenna design specifications (according to Figure 11).

Parameter	Dimension (mm)	Parameter	Dimension (mm)
W	19.36	W_4_	4.676
L	27.72	L_2_	2.695
g	1.32	W_5_	3.575
W_1_	3.63	L_3_	3.52
W_2_	2.78	L_g_	27.72
W_3_	3.708	W_6_	3.3
L_1_	9.51	W_g_	19.36

**Figure 10:**
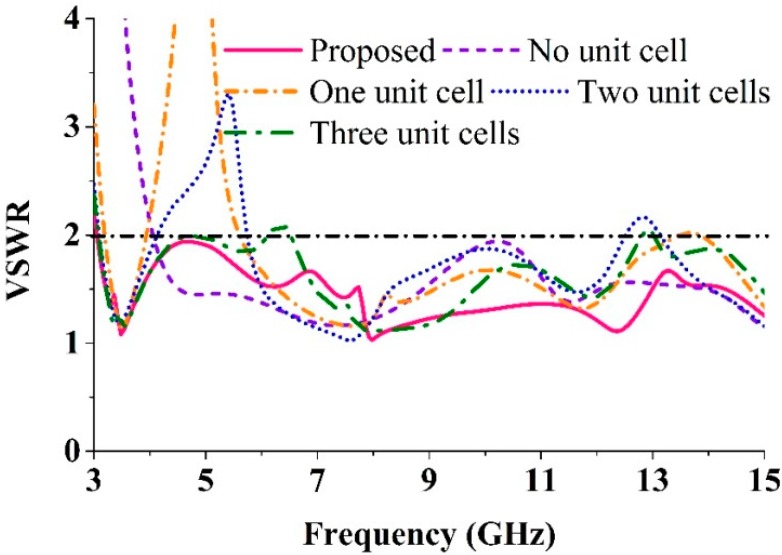
The effects of the unit cell of the patch on the VSWR.

The effects on the VSWR of using unit cells on the radiation patch are shown in Figure 10. The results of a proper analysis supports the use of four unit cells. It can be observed from Figure 10 that the proposed antenna design with four unit cells has the optimal computed results with respect to VSWR while also covering the standard UWB frequency range (3.1–10.6 GHz), even if size is reduced.

**Figure 11:**
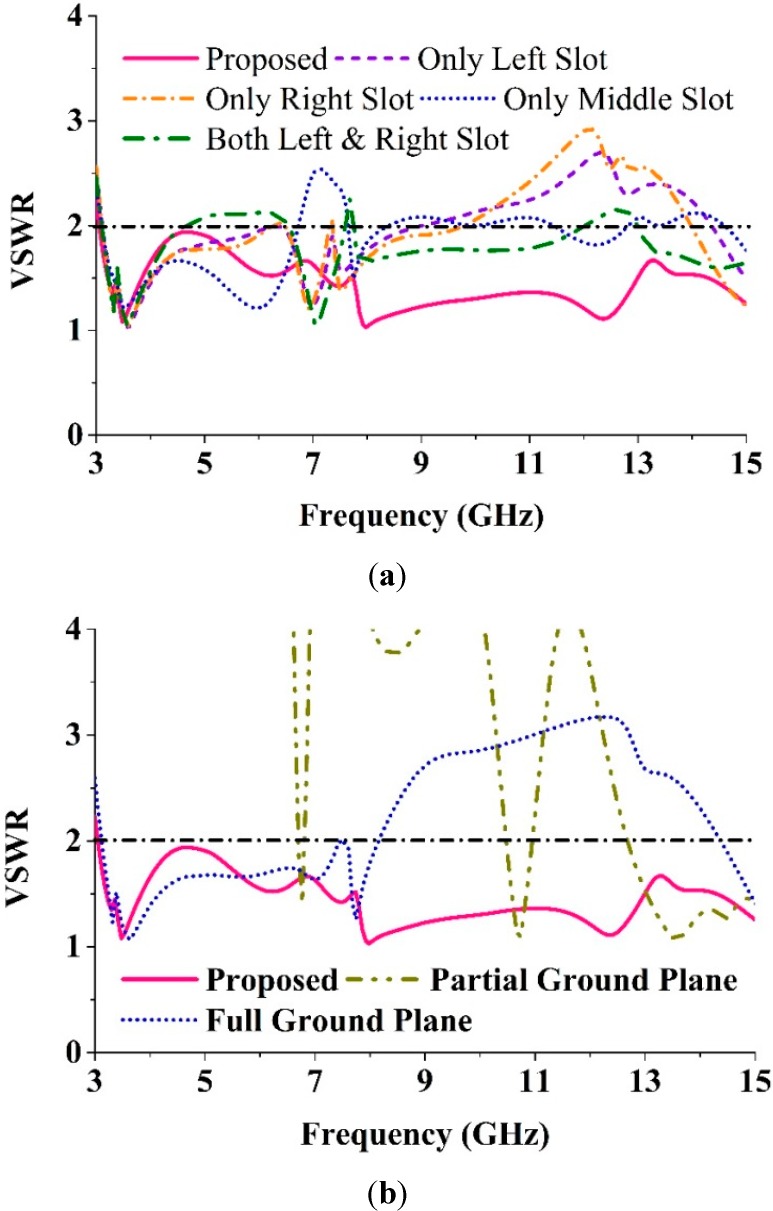
(**a**) The effects of various slots on the ground plane; (**b**) The effects of the ground plane size.

Figure 11a shows the effect on the VSWR of various slots on the ground plane. It can be seen that for the proposed antenna, the best results within the operating UWB frequency band were obtained for three slots used on the ground plane. Figure 11b compares the VSWRs of a full ground plane, a partial ground plane, and the proposed ground plane. It can be seen from Figure 11b that the ground plane of the proposed antenna delivers the optimal VSWR.

## 5. Experimental Validation 

**Figure 12:**
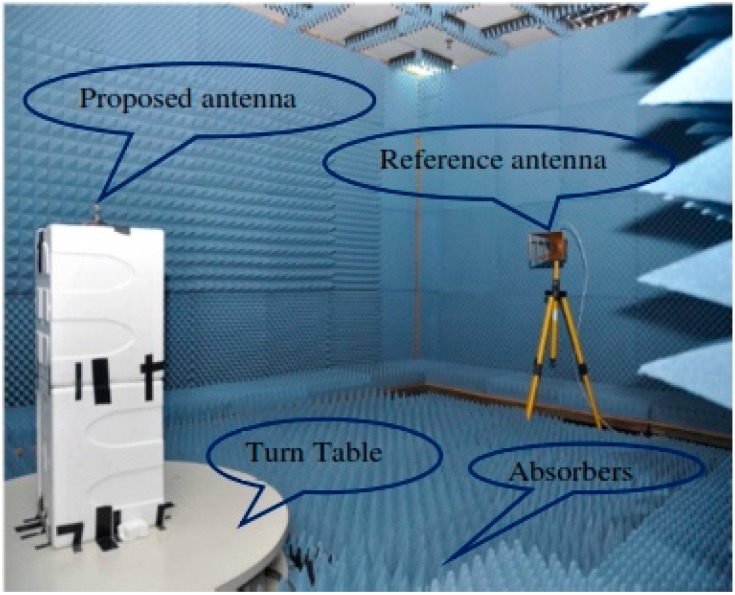
The anechoic chamber for the proposed UWB antenna.

**Figure 13:**
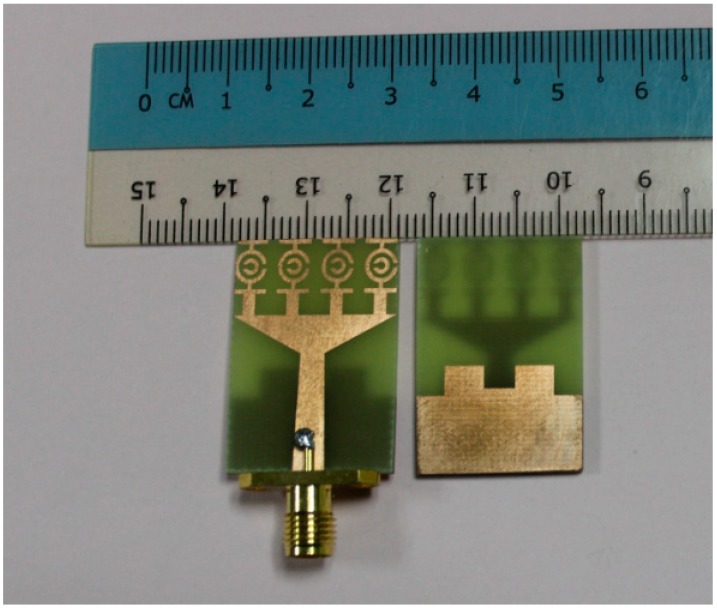
Photograph of the fabricated UWB MTM antenna.

**Figure 14:**
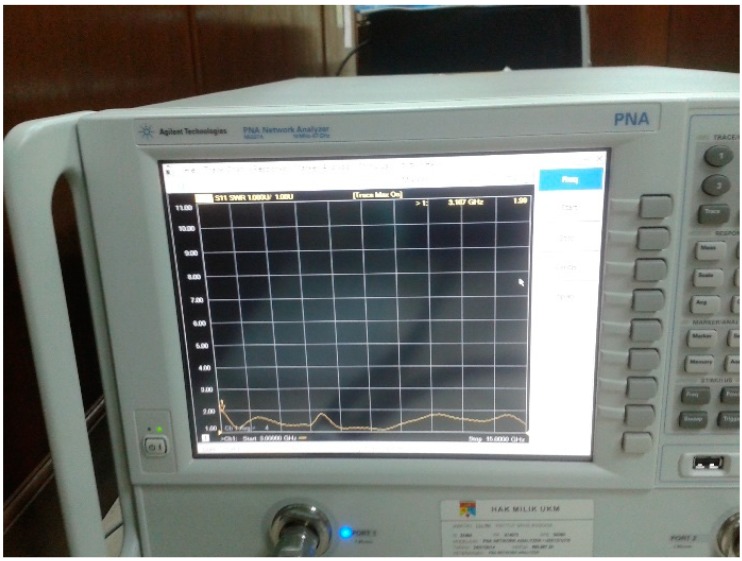
Agilent N5227A PNA Network Analyzer.

**Figure 15:**
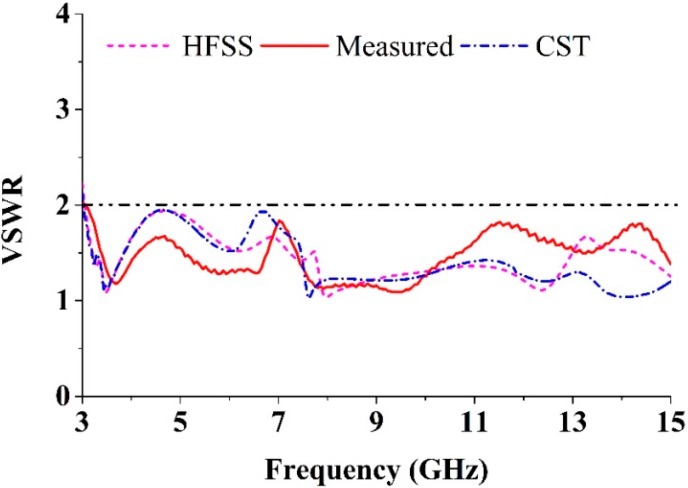
Comparison between simulated and measured VSWR.

Using a finite element method (FEM) based on the high frequency electromagnetic simulator HFSS and the CST simulator software, the performance of the proposed UWB antenna was analyzed, optimized, and plotted using the scientific graphing and data analysis software OriginPro. The proposed MTM antenna was prototyped on the PCB LPKF (S63) prototyping machine to obtain a physical test model. An anechoic chamber enabled an effective electromagnetic measurement system. The proposed SWB antenna prototype was tested in a rectangular-shaped anechoic chamber with dimensions 5 m × 5 m × 3 m. A double ridge guide horn antenna was adopted as a reference antenna. During measurement, the prototype antenna was oriented face to face with respect to the reference antenna. Figure 12 shows a photograph of the anechoic chamber. A pyramidal-shaped and electrically thick foam absorber was adopted on the wall, ceiling, and floor with less than −60 dB reflectivity at normal incidence. A turn Table 1 m in diameter, connected with a cable 10 m long among the controllers, rotated the test antenna with a rotation angle of 360° and a rotation speed of 1 rpm. A photograph of the fabricated antenna is shown in Figure 13. Measurements taken using a N5227A PNA network analyzer (10 MHz–67 GHz) are shown in Figure 14. The simulated and measured VSWR curves for the antenna are shown in Figure 15. The simulated impedance bandwidth covers the frequency range spanning from 3.06–15 GHz in HFSS and 3.03–15 GHz in the CST software. The measured bandwidth covers frequency range spanning from 3.1 to 15 GHz, which is good agreement with the simulated results.

**Figure 16:**
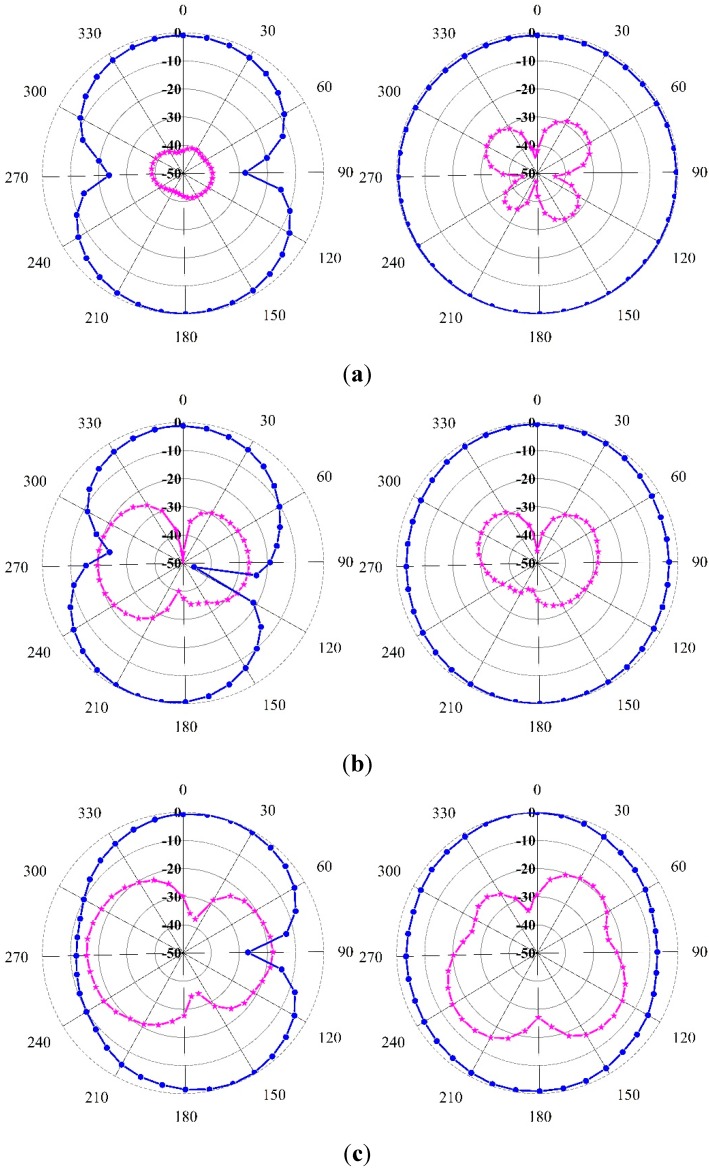

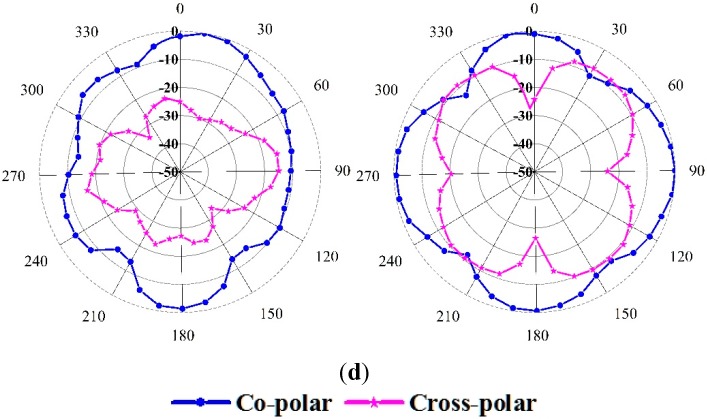
The measured radiation pattern at (**a**) 4 GHz; (**b**) 6 GHz; (**c**) 8 GHz; (**d**) 10 GHz.

The measured normalized radiation pattern of the proposed antenna is shown in Figure 16 for (a) 4; (b) 6; (c) 8; and (d) 10 GHz using both E-plane and H-plane. Two-dimensional (2D) radiation patterns are shown to indicate cross and co-polarization. To denote the co-polar and cross-polar, *E*_𝜃_ and *E_𝜑_* are plotted, respectively, where the x-z plane is considered as the H-plane and the y-z plane is considered as the E-plane. Cross-polarization was lower than co-polarization, which is characteristic of a standard radiation pattern. It was observed from the pass-band frequencies at 4, 6, 8 and 10 GHz that the proposed antenna exhibited better broadside radiation features (the antenna bandwidth operated at the frequency range over which the power density of the broadside radiation was within 3 dB of the maximum.) and considerable front-to-back ratio with low cross polarization, resulting in a symmetrical and nearly omni-directional radiation pattern. The proposed antenna exhibited linear polarization, because the level of cross-polarization was less than the level of co-polarization in the radiation pattern. The radiation pattern was found have some additional desirable characteristics. One was that the radiation pattern was more stable over the frequencies covered. Resonances did not shift abruptly with direction, so that a stable amount of power existed in the broadside beam. The cross-polarization was comparatively higher in the radiation pattern, possibly because of diffractions from the edges of the ground plane and the patch. These radiation patterns are appropriate for UWB applications.

**Figure 17:**
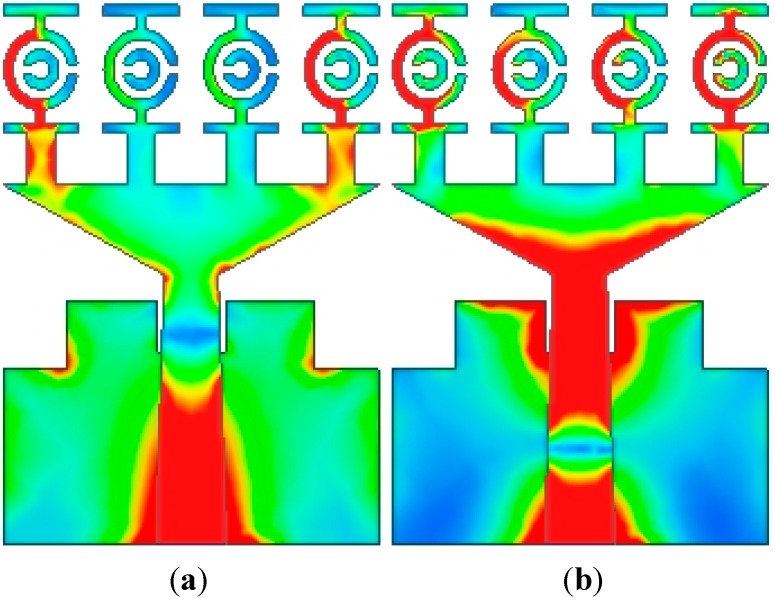

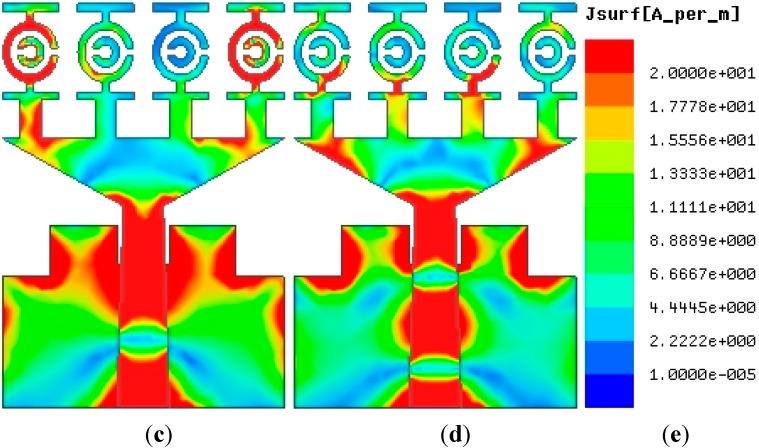
The surface current distribution at (**a**) 4 GHz; (**b**) 6 GHz; (**c**) 8 GHz; (**d**) 10 GHz; (**e**) Scale.

The surface current distribution of the proposed MTM UWB antenna at frequencies 4, 6, 8 and 10 GHz are shown in Figure 17. It is observed that the unit cells on the patch, microstrip line and rectangular slots at the top edge of the partial ground plane played important roles for creating resonances and achieving UWB frequency bands with a negative index metamaterial. In particular, the microstrip line and slotted ground plane had major effects at 4, 6, 8 and 10 GHz. At the top edge on the patch, there were strong effects due to the 1st and 4th unit cells at 4 GHz; 1st , 2nd, 3rd, and 4th unit cells at 6 GHz; 1st, and 4th unit cells at 8 GHz; and 1st, 2nd, 3rd unit cells at 10 GHz. This ensures that the performance of this UWB antenna depends on the unit cells on the patch, the feeding, and the rectangular slots at the top edge on the partial ground plane. The surface current maintains a harmonic flow at both the patch and the ground plane, resulting in the generation of an ultra-wide frequency band by a negative index metamaterial.

**Figure 18:**
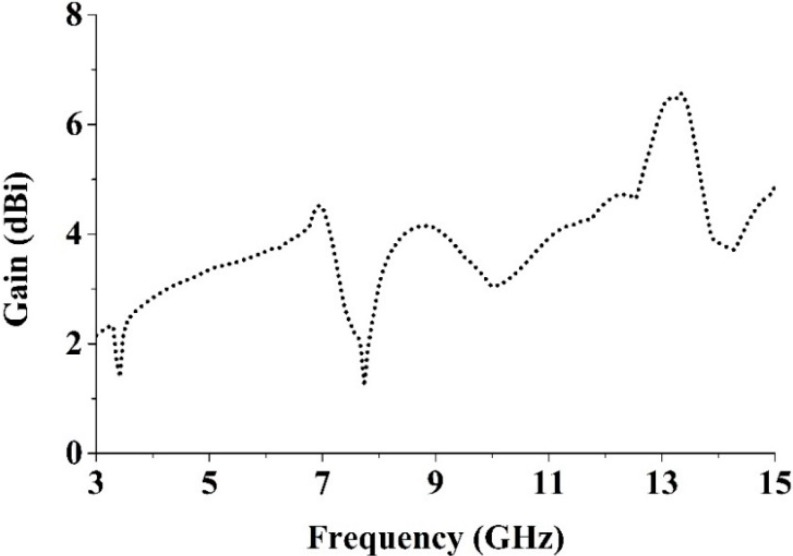
The measured peak gain of the proposed UWB MTM antenna.

The measured gain of the proposed UWB antenna is shown in Figure 18. A standard three-antenna system was used for measuring the gain with two identical horn antennas. The gains of the two identical horn antennas are known and obey well-known equations that are applied to the case of three antennas. The gain of the antenna under test can be calculated using the following equations, where *Pr* is the radiated power, and *R* is the distance between the two antennas under consideration.

For Antenna 1 (horn) and Antenna 2 (horn):

(10) $$G_{1}+G_{2}=20log_{10}\left(\frac{4\pi R}{\lambda }\right)+10log_{10}\left(\frac{P_{r2}}{P_{r1}}\right)$$

For Antenna 1 (horn) and Antenna 3 (under test):

(11) $$G_{1}+G_{3}=20log_{10}\left(\frac{4\pi R}{\lambda }\right)+10log_{10}\left(\frac{P_{r3}}{P_{r1}}\right)$$

For Antenna 2 (horn) and Antenna 3 (under test):

(12) $$G_{2}+G_{3}=20log_{10}\left(\frac{4\pi R}{\lambda }\right)+10log_{10}\left(\frac{P_{r3}}{P_{r2}}\right)$$

For directivity D, the following equation [35] is used where U is the radiation intensity and *Prad* is the total radiated power:

(13) $$D=\frac{4\pi U}{P_{rad}}$$

**Figure 19:**
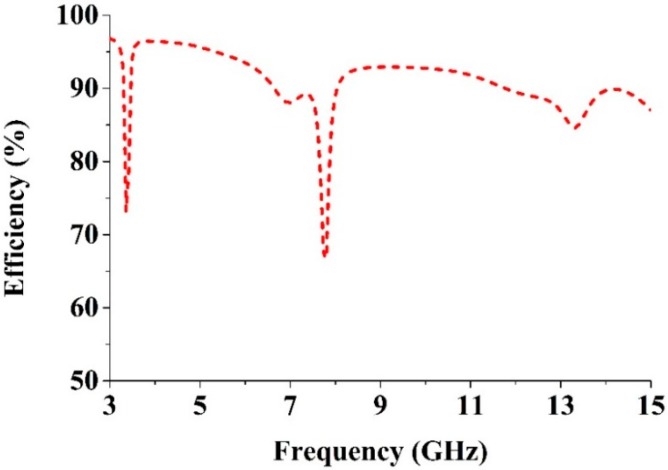
The radiation efficiency of the reported UWB antenna.

From Figure 19, it is seen clearly that the average gain of the proposed UWB antenna is 3.81 dBi, the maximum peak gain is 6.57 dBi, and the minimum gain is 1.22 dBi, which are acceptable for UWB operation.

The radiation efficiency of the proposed antenna is shown in Figure 19. It is seen that the average radiation efficiency is 91%, the maximum efficiency is 96.80% and the minimum efficiency is 66.75%. Table 4 compares the proposed UWB antenna and existing antennas.

**Table 4:** Comparisons between the proposed UWB antenna and existing antennas.

Antennas	Application	BW GHz (−10 dB)	Dimension (mm^2^)	Electrical Dimension	FBW (%)	Gain dBi
[23]	Medical Imaging	3.10–11.00	50 × 50	0.52 λ × 0.52 λ	112.01	4.3~10.8
[25]	Ultra-Wideband	5.20–13.90	25 × 25	0.43 λ × 0.43 λ	91.01	1.2~3.85
[26]	Microwave Sensing	2.70–9.70	22.25 × 20	0.20 λ × 0.18 λ	112.90	not reported
[27]	Ultra-Wideband	2.90–9.90	22 × 21	0.21 λ × 0.20 λ	109.38	−1.0~5.0
[28]	Microwave Imaging	3.80–11.85	30 × 30	0.38 λ × 0.38 λ	102.00	not reported
[29]	Microwave Imaging	1.15–4.40	75 × 75	0.29 λ × 0.29 λ	117.12	2.0~8.0
[30]	Microwave Imaging	4.00–9.00	30 × 30	0.40 λ × 0.40 λ	76.92	2.0~6.0
[31]	Microwave Imaging	3.40–12.50	16 × 21	0.18 λ × 0.24 λ	114.50	1.0~5.16
Proposed	Microwave Sensing	3.10–15.00	19.36 × 27.72	0.20 λ × 0.29 λ	131.50	1.2~6.57

The proposed antenna and the existing antennas [23,25,26,27,28,29,30,31] were also studied to ensure an impartial comparison where all reference antennas cover ultra-wideband spectrum reported in the literature review. The performances parameters, such as applications, 10-dB bandwidth, dimensions, electrical dimensions, fractional bandwidth and gain were discussed. Although the proposed antenna may not have a better gain than of the references [23,29], a good fractional bandwidth (FBW, 131.50%) with a smaller electrical dimension was pointed out. Therefore, the proposed UWB metamaterial antenna can offer good compact characteristics while maintaining much smaller dimensions than the designs in [23,28,29,30]. Table 4 summarizes the comparisons between the proposed UWB antenna and existing antennas.

## 6. Time Domain Performance

**Figure 20:**
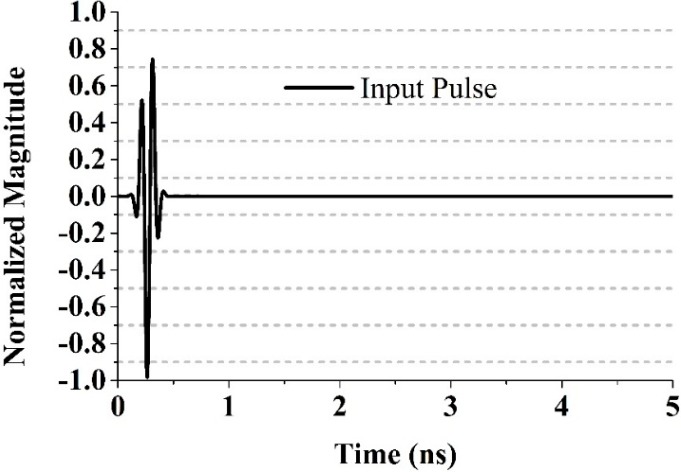
The input pulse with τ = 67 ps.

A full-wave simulation using CST Microwave Studio was conducted to observe the time domain performance of the proposed SWB antenna. The analysis followed the methods explained in [31,36,37,38]. A fourth-order Rayleigh pulse was used as the input pulse which takes the following form (Figure 20):

(14) $$s(t)=[\frac{12}{\tau^{2}}-\frac{48}{\tau^{6}}{\left(t-1\right)}^{2}+\frac{16}{\tau^{8}}{\left(t-1\right)}^{4}]\exp(-{\left(\frac{t-1}{\tau }\right)}^{2})$$

**Figure 21:**
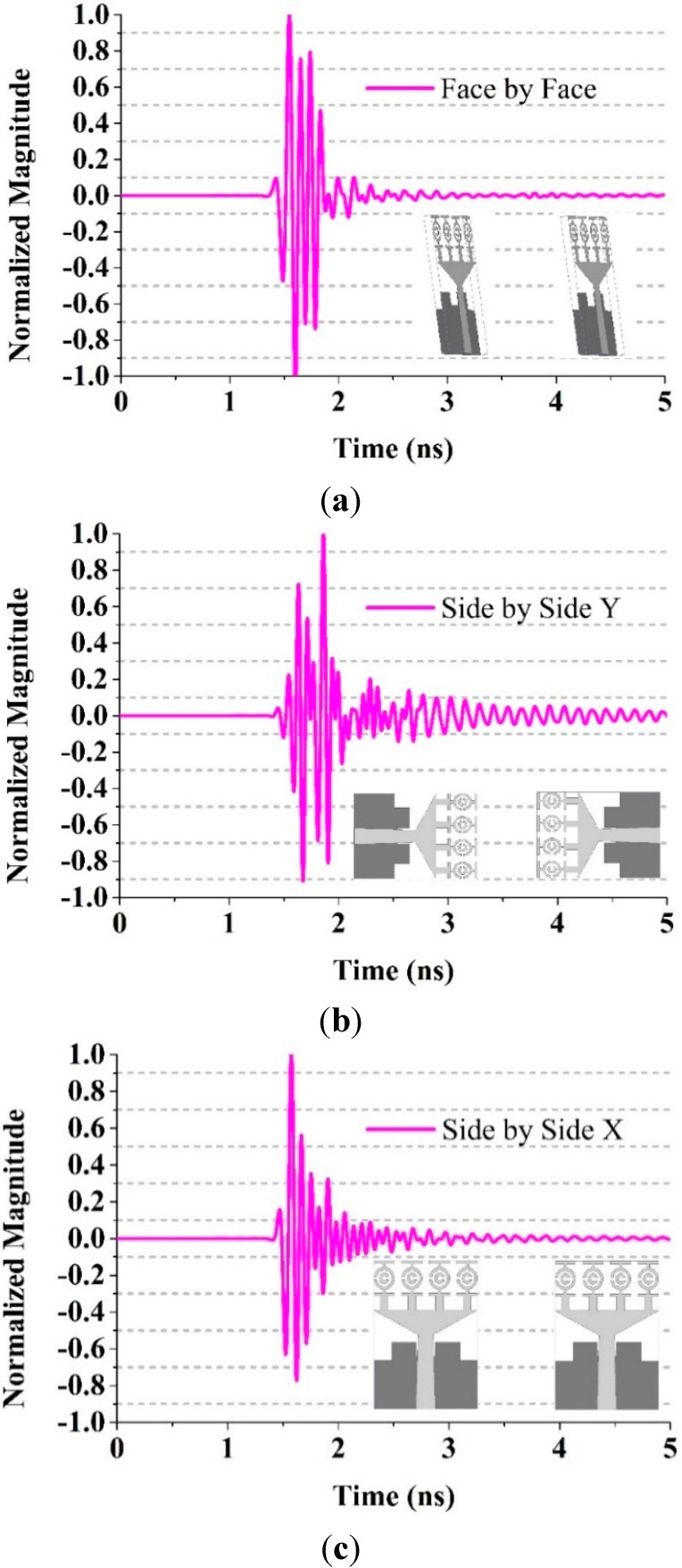
Pulse transmission analysis in different orientation of the proposed fractal UWB antenna. (**a**) Face-to-face; (**b**) Side-by-side Y; (**c**) Side-by-side X.

The correlation coefficient between the received signal and the transmitted signal can indicate the extent of pulse distortion that occurred during conduction through the antenna. The fidelity factor (F) can be calculated by applying Equation (15) [31,37]:

(15) $$F=\max_{\tau }\left|\frac{\int_{-\infty }^{+\infty }s(t)r(t-\tau )}{\sqrt{\int_{-\infty }^{+\infty }s{\left(t\right)}^{2}dt.\int_{-\infty }^{+\infty }r{\left(t\right)}^{2}dt}}\right|$$

where, the transmitted signal is denoted by *s*(*t*) and the received signals by *r*(*t*). Having a high degree of correlation between the received and transmitted signals is necessary in UWB impulse radio communications for avoiding the loss of the modulated information. However, the fidelity factor is less important for most of the other types of telecommunication systems. The time domain characteristics of the reported UWB antenna was also simulated. Three configurations were chosen: side-by-side in the Y direction, face-to-face, and side-by-side in the X direction. A narrow pulse was delivered at a distance 300 mm from the transmitter, and the received pulse was calculated. Figure 21 shows the pulse transmission analysis of different orientations of the proposed antenna. 

The transmitted pulse and the received pulse are normalized with respect to the maximum level. The figure shows a pulse with negligible distortion in terms of the peak value (unity). The fidelity factor were 91%, 87% and 84% when the antenna was configured face-to-face, side-by-side in the Y direction, and side-by-side in the X direction, respectively. Ideally, the antenna favors a distortionless narrow pulse for proper function.

**Figure 22:**
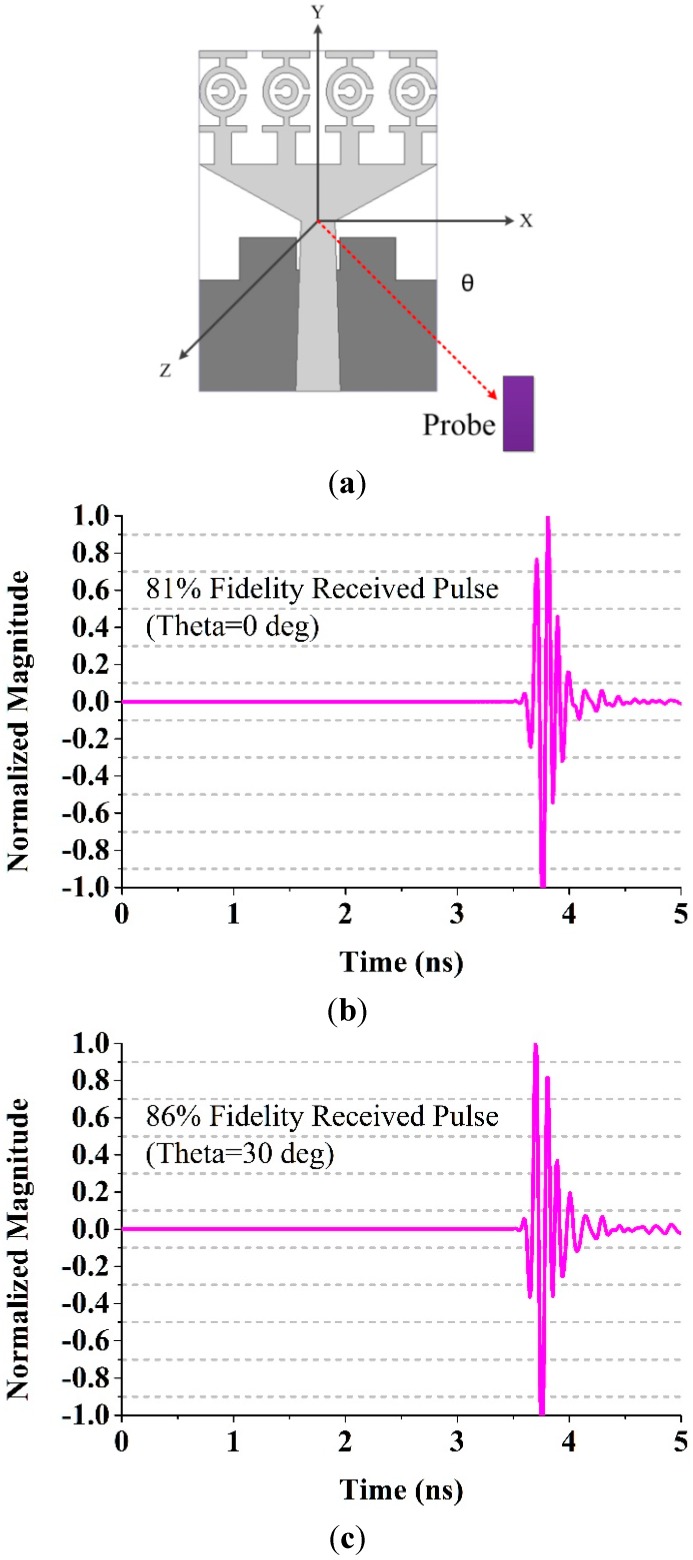

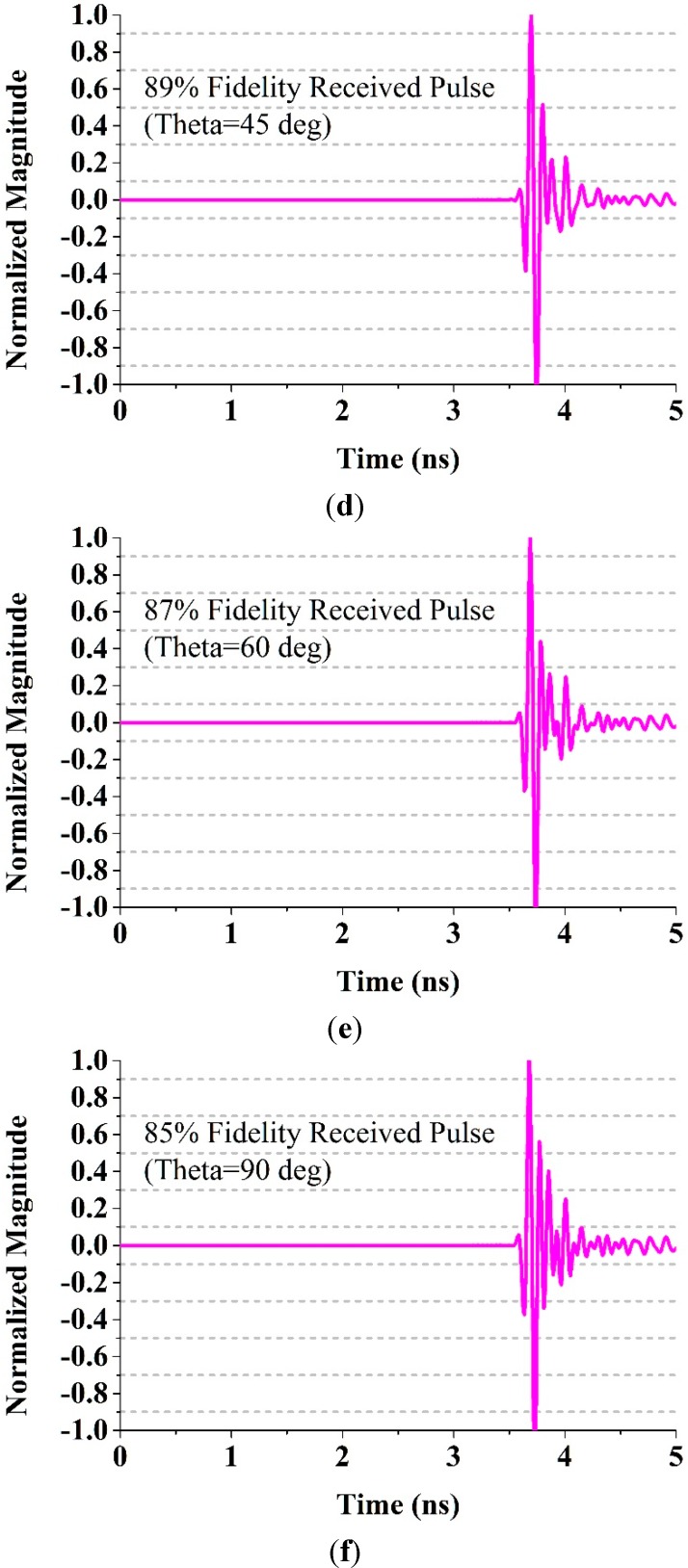
Normalized received signals by virtual probe for Phi = 90° and varying Theta in the E-plane, (**a**) Antenna with probe; (**b**) Theta=0°; (**c**) Theta = 30°; (**d**) Theta = 45°; (**e**) Theta = 60°; (**f**) Theta = 90°.

We also conducted a simulation similar to that of [38] in which a transmitting antenna and five virtual probes were installed in the configuration illustrated in Figure 22. The probes were placed in the x-z plane with theta equal to 0°, 30°, 45°, 60° and 90°. The distance between the transmitting antenna and the probes was 1 m. The calculated fidelity factors are 81%, 86%, 89%, 87% and 85% for each value of theta, respectively.

## 7. Imaging Results and Discussion 

**Figure 23:**
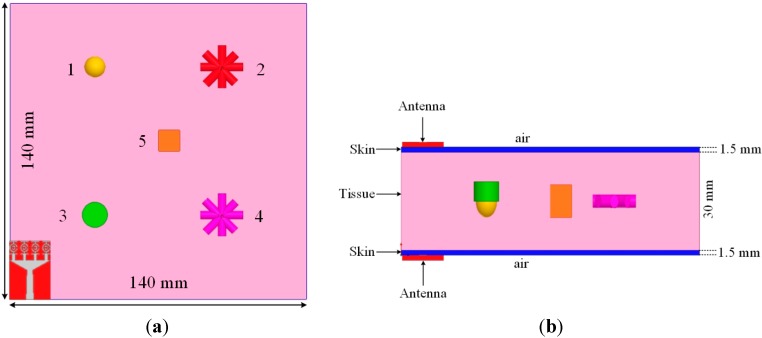
The breast phantom with tumour simulants (**a**) Top view; (**b**) Cross view including two proposed UWB antennas.

**Figure 24:**
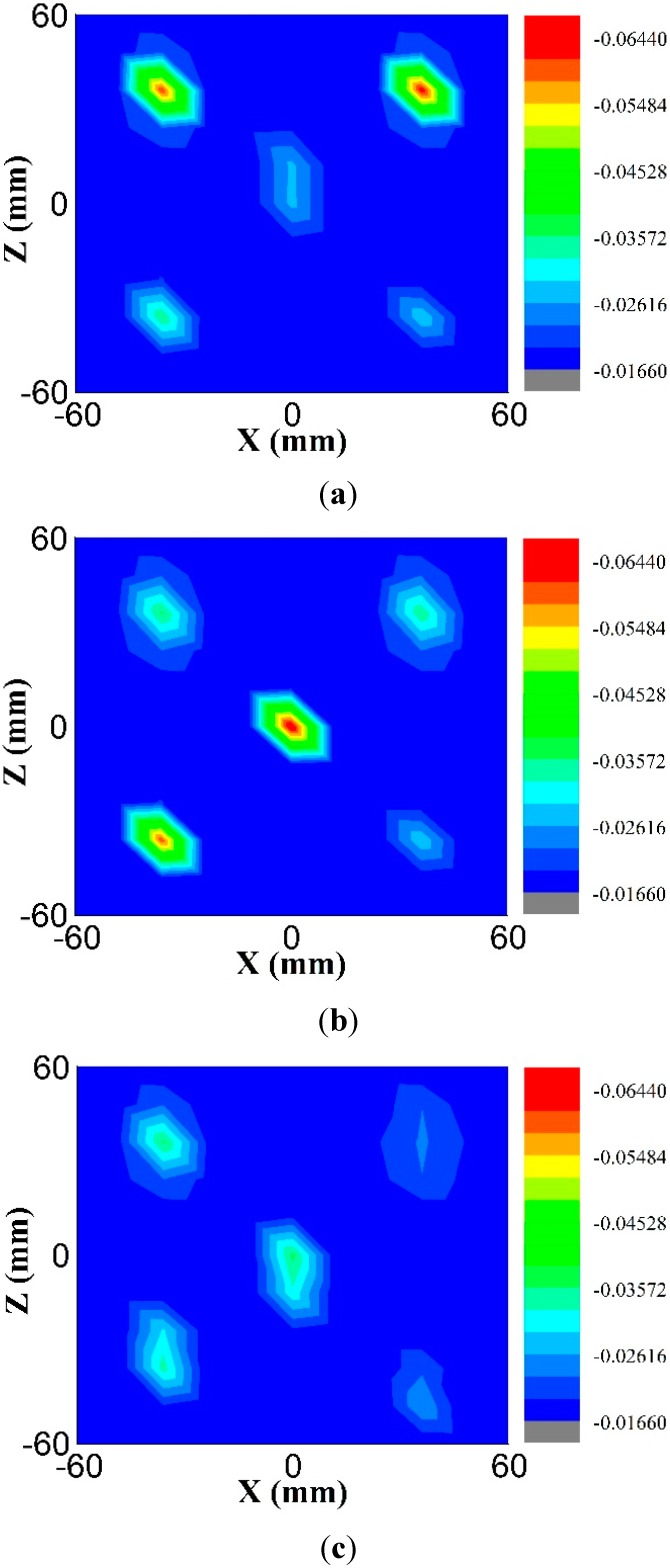
The simulated $\left|S_{21}^{cal}\right|$ images from 2-D scanning at (**a**) 5.2 GHz; (**b**) 6.9 GHz; (**c**) 8.8 GHz.

The proposed antenna performance as a microwave imaging sensor was tested using an aperture scanning method [39] to detect tumors in a breast phantom (lossy dielectric). The breast phantom belonged to a two-layer medium with a tissue layer and a skin layer. The tissue layer and skin layer were made with thickness of 30 mm and 1.5 mm, respectively. The properties of these layer were discussed in [39,40,41]. The breast phantom had dimensions 140 mm × 140 mm with five tumor simulants (Figure 23a). Tumor 1 had a spherical shape (radius 4 mm), Tumor 3 had a cylindrical shape (radius 6 mm and height 6 mm), and Tumor 5 had a cuboid shape (side length 10 mm); Tumor 2 and 4 had irregular shapes (radius 2 mm and height 20 mm). The permittivity and conductivity of these tumors were 67 and 5 S/m, respectively. As shown in Figure 23b, the breast tissue was covered with two layers, one at the top portion and one at the bottom portion, which were scanned with two antennas (one transmitting and the other receiving). The following Equation (16) was adopted for imaging [39]:

(16) $$S_{21}^{\mathrm{cal}}(x,z)=S_{21}^{\mathrm{meas}}(x,z)-S_{21}^{\mathrm{back}}(x,z)$$

where $S_{21}^{back}(x,z)$ is the transmission S-parameter between two the UWB antennas where only the background medium is presented (no scattering) whereas $S_{21}^{\mathrm{cal}}(x,z)$ is the calibrated transmission S-parameter for any target, and $S_{21}^{\mathrm{meas}}(x,z)$ is the measured transmission S-parameter. The images were constructed from the plots of $\left|S_{21}^{cal}(x,z)\right|$. The potential of using the reported antennas for this scanning method was investigated using the EM solver CST. The transmission S-parameter (S_21_) was obtained on an area of 120 mm × 120 mm with a 10 mm spatial sampling rate. The images acquired from $\left|S_{21}^{cal}\right|$ at 5.2 GHz, 6.9 GHz and 8.8 GHz are shown in Figure 24. Tumor simulants were detected easily at 5.2 GHz and 6.9 GHz, but their shapes were not clearly identifiable because they were electrically too small.

## 8. Conclusions

A negative index metamaterial-inspired UWB antenna has been presented with an integration of complementary SRR and CLS unit cells to investigate its tumour detection capability for microwave imaging sensor applications. The entire design procedure for the antenna has been detailed. The proposed antenna sensor has several advantages: (i) direct contact with the imaged body; (ii) ultra-wideband performance; (iii) easy fabrication, (iv) compact size, and (v) high fidelity factor. The fabricated MTM antenna sensor delivers 131.5% fractional bandwidth covering the frequency band spanning from 3.1–15 GHz (VSWR < 2) a maximum radiation efficiency of 96.80% and maximum gain of 6.57 dBi. The fidelity factor has been calculated for various antenna sensor orientations and various probes. The performance of the proposed antenna has been tested using an aperture scanning method to detect tumours in a lossy dielectric breast phantom. The advantages of the proposed MTM UWB antenna, including the capability to detect tumour simulants, high fidelity factor and gain, smooth surface current distribution and nearly omni-directional radiation patterns with low cross-polarization confirm that the antenna is appropriate for microwave imaging sensor applications.
